# Endophytic Fungi of Native *Salvia abrotanoides* Plants Reveal High Taxonomic Diversity and Unique Profiles of Secondary Metabolites

**DOI:** 10.3389/fmicb.2019.03013

**Published:** 2020-01-17

**Authors:** Yeganeh Teimoori-Boghsani, Ali Ganjeali, Tomislav Cernava, Henry Müller, Javad Asili, Gabriele Berg

**Affiliations:** ^1^Department of Biology, Faculty of Science, Ferdowsi University of Mashhad, Mashhad, Iran; ^2^Institute of Environmental Biotechnology, Graz University of Technology, Graz, Austria; ^3^Department of Pharmacognosy, Faculty of Pharmacy, Mashhad University of Medical Sciences, Mashhad, Iran

**Keywords:** *Salvia abrotanoides*, endophytic fungi, secondary metabolites, cryptotanshinone, gibberellin

## Abstract

Endophytic fungi are often embedded in their host’s metabolic networks, which can result in alterations of metabolite production and higher amounts of active compounds in medicinal plants. This study reports the occurrence, diversity, and secondary metabolite profiles of endophytic fungi isolated from *Salvia abrotanoides* plants obtained from three geographically distinct sites in Iran. A total of 56 endophytic fungi were isolated from roots and leaves of *S*. *abrotanoides;* site-specificity and root-dominated colonization was found to be a general characteristic of the endophytes. Based on molecular identification, the endophytic fungi were classified into 15 genera. Mycelial extracts of these isolates were subjected to high-resolution mass spectrometry analyses and revealed a broad spectrum of secondary metabolites. Our results demonstrated that *Penicillium canescens*, *P. murcianum*, *Paraphoma radicina*, and *Coniolariella hispanica* are producers of cryptotanshinone, which is a main bioactive compound of *S*. *abrotanoides*. Moreover, it was shown that it can be produced independent of the host plant. The effect of exogenous gibberellin on *S. abrotanoides* and endophytic fungi was shown to have a positive effect on increasing the cryptotanshinone production in the plant as well as in endophytic fungi cultivated under axenic conditions. Our findings provide further evidence that endophytic fungi play an important role in the production plant bioactive metabolites. Moreover, they provide an exploitable basis to increase cryptotanshinone production in *S*. *abrotanoides*.

## Introduction

Plants can be considered as holobionts that are embedded in multiple mutualistic networks connecting them with the environment and microbial communities of varying structure and diversity (Vandenkoornhuyse et al., 2015). Plant-microbe interactions can be very profound and versatile, especially with highly adapted endophytes (Hardoim et al., 2015). Plant endophytes can improve their host’s resistance against biotic and abiotic stress by provision of various bioactive compounds (Gunatilaka, 2006). Previous studies have shown that endophytic microbial communities within medicinal plants have a great potential as producers of novel bioactive compounds and thus have a high potential for agricultural, pharmaceutical, and other applications (Köberl et al., 2013; Rai et al., 2014). Moreover, it is known that endophytes can produce distinct host plant metabolites or their precursors; e.g., taxol (Stierle et al., 1993), comptothecin (Kusari et al., 2009), azadirachtin (Kusari et al., 2012), tanshinone I/IIA (Ming et al., 2012), and maytansine (Wings et al., 2013). In addition, microbial inoculants can enhance the concentration of bioactive metabolites in medicinal plants as shown for *Bacillus subtilis* Co1-6 and *Paenibacillus polymyxa* Mc5Re-14, which enhanced apigenin-7-O-glucoside in chamomile (Schmidt et al., 2014), and *Chaetomium globosum* D38, which promotes bioactive constituent accumulation and root production in *Salvia miltiorrhiza* (Zhai et al., 2018). Despite the evident potential to improve the availability of active compounds for diverse health issues, plant-endophyte interactions and their metabolic interplay in medicinal plants are not yet fully understood.

*Salvia abrotanoides* (Kar.) Sytsma, which is part of the *Lamiaceae* family, was previously also known as *Perovskia abrotanoides* Kar. (Drew et al., 2017). It is a traditional medicinal plant, growing in various regions of Iran. This plant grows as a bush or semi-shrub with a height of about one meter and is propagated by seeds (Ghahreman, 1993). The roots of this rather unknown medicinal plant are mainly used for the treatment of leishmaniasis in Iranian folk medicine (Jaafari et al., 2007). There are some reports that imply leishmanicidal, antiplasmodial, anti-inflammatory, antibacterial, and cytotoxic pharmacological effects of *S. abrotanoides* (Hosseinzadeh and Amel, 2001). These effects are attributed to the presence of tanshinones as the most important and most abundant bioactive compounds in the roots of this plant (Sairafianpour et al., 2001). Although not all relevant biosynthetic pathways have been explored in detail, some common key enzymes were previously described (Hedden et al., 2001). Tanshinones are abietane-type norditerpenoid quinones that were first identified in 1930s from the roots of *Salvia miltiorrhiza* (Nakao and Fukushima, 1934). For this compound group, diverse pharmacological activities such as anticancer (Hu et al., 2015), antidiabetes (Kim et al., 2007), cardioprotective effects (Fu et al., 2007) and neuro-protective activity (Yu et al., 2007) have been reported. Moreover, cryptotanshinone as a prominent member of tanshinones is known for its antibacterial activity (Cha et al., 2013) and strong anticancer properties (Hu et al., 2015; Li et al., 2015; Wu et al., 2016). The effectiveness and potential usefulness of tanshinones led to a number of studies with the aim of increasing their concentration *in planta* by different approaches. Some of these studies explored the effects of biotic and abiotic elicitors on improvement of the accumulation of tanshinons in plants (Hao et al., 2015; Zaker et al., 2015). Recently, the implementation of endophytic microorganisms in order to discover novel, biologically active compounds was expanded (Bedi et al., 2018; Sharma et al., 2018). However, nothing is known about the occurrence, diversity and secondary metabolite profiles of endophytic fungi in *Salvia abrotanoides* and microbial producers of tanshinones.

In the present study, 56 endophytic fungal strains were isolated from native *S. abrotanoides* plants grown in different arid areas in Northern Iran to specifically screen for potential producers of tanshinones. In addition, an untargeted metabolite profiling approach was implemented in order to characterize isolates that produce a high diversity of secondary metabolites. Such isolates can serve as a valuable bioresource in the future to increases the concentration of distinct compounds *in planta* or in biotechnological applications. In a complementary approach, we explored possibilities to improve the accumulation of tanshinones *in planta* and the development of cultivation methods for fungi with the same aim. We expected that addition of end products from interconnected biosynthetic pathways might have favorable effects on the production of cryptotanshinone due to regulation mechanisms. A positive effect of gibberellin supplementation on the cryptotanshinone biosynthesis in *S. abrotanoides* and in different endophytic fungi was discovered and therefore studied in more detail.

## Materials and Methods

### Sample Collection and Isolation of Endophytic Fungi

Endophytic fungi were isolated from the roots of *Salvia abrotanoides* (Kar.) in flowering stage. Plant sampling was conducted at three different locations in the northeast of Iran (Zoshk, N 36°16′58″, E 59°07′06″, Kalat, N 36°35′07″, E 59°52′12″, Darrud, N 36°10′57″, E 59°10′12″), and a voucher specimen was deposited with the herbarium of Ferdowsi University of Mashhad, Iran, under voucher code 36763 (FUMH). The samples were kept at 4°C and the isolation of fungal endophytes was conducted within 24 h after sample collection. For fungal isolation, all plant tissues were surface-sterilized using the procedure described by Fisher et al. (1993). The roots of plants were washed under running water and cut to 15 mm segments. Then the root segments were surface-sterilized by sequential immersion in 70% ethanol for 1 min, sterilized water for 1 min, 2.5% sodium hypochlorite for 3 min, sterilized distilled water for 1 min, and 70% ethanol for 30 s. The roots were then rinsed three times in sterilized water for 1 min to remove the remaining chemicals from their surface. Sterilization of leaves was conducted with analogous protocol as for the roots, where the sodium hypochlorite solution was diluted three times and the immersion time was reduced to 1 min. The effectiveness of the sterilization process, which resulted in the elimination of all epiphytic microorganisms was confirmed using aliquots of sterile, distilled water from the last rinse. Aliquots from all samples were inoculated on culture media in Petri dishes and checked for microbial growth. Two root segments were then evenly placed in Malt extract agar plates (30 g malt extract, 3 g soya peptone, 15 g agar, 1000 ml deionized water) augmented with 100 mg l^–1^ Streptomycin to avoid bacterial contamination. The Petri dishes were sealed with Parafilm (PM-996) and incubated at 25 ± 2°C in an incubator until the fungal colonies emerged from root sections. Hyphal tips of fungal colonies were transferred to new Petri dishes with malt extract agar (MA) to obtain pure cultures of the fungal isolates.

### DNA Extraction, Amplification of the ITS Region, and Sequencing

A molecular approach was implemented to identify the fungal isolates. The endophytic fungi were grown on fresh MA medium for 5–7 days. Mycelium of each isolate was then transferred into a tube with glass beads (250 mg of beads with a diameter of 0.25–50 mm and two beads of 2.85–3.45 mm) and 450 μl of DNA extraction buffer (200 mM Tris–HCl, 250 mM NaCl and 0.5% SDS). The mechanical disruption of mycelia was performed by shaking 2 × 30 sec in a FastPrep intstrument (MP Biomedicals, Solon, OH, United States). Subsequently, fungal DNA was extracted with the phenol/chloroform method. In the final step the ethanol was decanted and the DNA-containing pellet was dried and resuspended in 50 μl of nuclease-free H_2_O. DNA quality and quantity were checked by spectrophotometry using a UV-Vis spectrophotometer (NanoDrop 2000c; Thermo Fisher Scientific, Waltham, MA, United States) and stored at −20°C until further processing. PCR amplification with genomic DNA from each isolate was performed in a 30 μl of PCR reaction mix with 1.8 μl MgCl_2_ (25 mM), 6 μl Taq-&Go, 0.6 μl of ITS1f primer (CTT GGT CAT TTA GAG GAA GTA A), 0.6 μl of ITS4r primer (TCC TCG GCT TAT TGA TAT GC), 20 μl PCR grade water, and 1 μl of the DNA template (White et al., 1990). The amplification was conducted with an initial denaturation at 95°C for 5 min, followed by 36 cycles of 95°C for 30 s, 54°C for 35 s, and 72°C for 40 s with a final extension at 72°C for 10 min using the TPersonal Combi, Biometra Thermocycler (Biometra GmbH, Germany). PCR products were purified using the Wizard SV Gel and PCR Clean-Up System (Promega, Madison, WI, United States) and quantified on a Nanodrop 2000c spectrophotometer (Thermo Fisher Scientific, Waltham, MA, United States). Subsequently, 14 μl of 20 ng μl^–1^ PCR product including one specific primer (ITS1f) was sent to LGC Genomics (Berlin, Germany) for sequencing. Sequences were identified using the BLAST algorithm against the NCBI Targeted Loci Nucleotide BLAST – Internal transcribed spacer region (ITS) database and were deposited in GenBank with the accession numbers MK367721- MK367776.

### Extraction of Secondary Metabolites From Fungal Isolates

Fungal mycelial plugs were used to inoculate 100 ml potato extract glucose broth (Carl Roth GmbH, Germany) in 250-ml Erlenmeyer flasks. The flasks were incubated in the dark at 30°C for 24 days with rotary shaking at 105 rpm. The fungal cultures were vacuum filtered through filter paper (Rotilabo^®^ round filter, type 112 A, 47 mm, Carl Roth GmbH, Germany) to remove the biomass. The mycelia were dried in an oven at 40°C to obtain the dry weight. The dried mycelia were then homogenized by mortar and pestle, suspended in 0.5 ml ethyl acetate and mechanically disrupted with glass beads (three beads with a diameter of 2.85–3.45 mm and 250 mg of 0.25–0.5 mm) for 2 × 40 s at 6 m/s in a FastPrep instrument (MP Biomedicals, Solon, OH, United States). Precooled ethyl acetate and glass beads at −70°C were used for reproducible extraction and to avoid further degradation of metabolites. The homogenate was centrifuged for 10 min, 13000 rpm at 4°C. Subsequently, 250 μl of each supernatant were collected and stored at 4°C before the HPLC-MS analysis was conducted.

### Detection of Bioactive Compounds by HPLC-MS Analysis

Extracts of fungal isolates were analyzed with a combined HPLC-hybrid quadrupole-orbitrap mass spectrometer (Q Exactive; Thermo Scientific, Bremen, Germany). Chromatographic separation of the extracts was performed on an Atlantis dc 18 μm, 2.1 × 100 mm column (Waters Corporation, United States) using H_2_O with 0.1% formic acid as solvent A and acetonitrile with 0.1% formic acid as solvent B with the following gradient elution program: at 0 min, the program started with 5% B and was increased to 40% B at 2 min, and subsequently to 100% B at 15 min, which was kept until the end of each run. The run time was 40 min at a solvent flow rate of 0.3 mL/min and a sample injection volume of 20 μl. Mass spectrometric detection was carried out in positive and negative mode using an electrospray ionization (ESI) source. The ESI conditions were set to 3.1 kV spray voltage and 330°C capillary temperature. Scans were recorded in the range 100.0–1500 m/z with the AGC target set to 1 × 10^6^ and maximal accumulation time of 200 ms. The resolution was adjusted to 70,000. Altering full MS-SIM and targeted MS^2^ cycles were employed and a specific inclusion mass of 297.14807 amu was selected. Standard calibration was obtained with 0.9, 1.8, 9.0, 13.5, 18, and 27 μg cryptotanshinone standard (Sigma-Aldrich, United States). The obtained mass spectra were analyzed with Compound Discoverer 2.0 (Thermo Scientific) and the integrated mzCloud database^1^ to detect secondary metabolites in an untargeted approach. For the initial analysis, the implemented HighChem HighRes algorithm was selected and the identity of the automatically detected compounds was confirmed by comparing their spectra with reference data available in the software package. Fungal isolates that produced either cryptotanshinone and/or ≥10 secondary metabolites that were detectable with the conducted experiment setup, were considered for further data visualizations and interpretations. For isolates assigned to the same fungal species, but originating from different isolation sites, the producer of the most diverse secondary metabolite profile was selected as the representative strain.

### Plant Material, Growth Conditions, and Gibberellin Treatments

Seeds of *S. abrotanoides* used in this experiment were collected in the Zoshk area, Iran. The seeds were sown in pots filled with a mixture of sandy soil, vermiculite and compost in a proportion of 3:1:1, respectively. The plants grown in a growth chamber (approximately 22/20°C day/night temperature, 55% relative humidity and a 15 h photoperiod) located at Graz University of Technology (Graz, Austria). A GA_3_ (Duchefa Biochemie BV, Haarlem, Netherlands) solution was applied three times with a weekly interval as foliar spray to the plants after 87 days, at concentrations of 0, 50, 100, and 150 mg l^–1^ in sterile water. All treatments were performed with low-pressure hand-wand sprayers and 5 ml for each pot. Sterile water without GA_3_ was implemented as a control. Following the growth period, the roots of plants grown in the same pots were separately dried in an oven at 35°C. Each of the treatments was conducted with three biological replicates. The roots were homogenized and subjected to extractions with the abovementioned method that was also used to extract metabolites from fungal samples.

### Cultivation of Selected Endophytic Fungi in Combination With Gibberellin Treatments

The isolates which were shown to produce cryptotanshinone were selected for gibberellin treatments in order to assess stimulatory effects. The isolates were cultivated in PGB medium and treated with 5 ml filtered (0.45 μm syringe filter) GA_3_ at a concentration of 50 mg l^–1^ in Erlenmeyer flasks (95 ml PGB and 5 ml GA_3_). Flasks including the fungus and 100 ml PGB medium were included as a non-treated control for each isolate. The flasks were kept in an incubation room at 21°C for 24 days shaking on a rotary shaker at 105 rpm. Each experiment was conducted with three biological replicates. The extraction of cryptotanshinone was conducted with the aforementioned method.

### Statistical Analysis

Statistical analyses were performed with SPSS v.20.0.0 (SPSS Inc., Chicago, IL, United States). Analysis of variance for plant treatments with gibberellin was performed with one-way ANOVA and the significance of the results was assessed with the Duncan *post hoc* test. The Univariate General Linear model and LSD test were implemented to assess the significance of the effects of gibberellin treatments on cryptotanshinone production in each fungal isolate. *P* values <0.05 were considered to be significant. All experiments were performed in triplicates (*n* = 3) and the results were reported as means ± standard error.

## Results

### Isolation and Identification of Endophytic Fungi

A total of 56 endophytic fungi were isolated from leave and root segments of *Salvia abrotanoides* at the three sites. Of these, only two isolates were recovered from the plant’s leaves (*Thielavia microspore* and *Aspergillus* sp.), while the remaining isolates were obtained from root samples. All isolates were grouped based on the site of sampling, and included the fungal genera *Penicillium*, *Paraphoma*, *Phaeoacremonium*, *Talaromyces*, *Aspergillus*, *Psathyrella*, *Trichoderma*, *Alternaria*, *Thielavia*, and *Acremonium* originating from Zoshk, *Fusarium*, *Talaromyces*, *Penicillium*, and *Coniolariella* from Kalat and *Fusarium*, *Paecilomyces*, *Simplicillium*, and *Monocillium* from Darrud (Table 1). Several of the isolates were represented by different species within the same genus. *Penicillium* was represented by four species including *Penicillium canescens, P. chrysogenum, P. charlesii* and *P. murcianum*. Moreover, one of the *Penicillium* isolates was not identifiable at the species level by the utilized molecular approaches. *Talaromyces* was represented by two different species including *Talaromyces verruculosus* and another species that was also not identified at the species level by molecular approach. In addition, the genus *Fusarium* was represented by two species including *Fusarium dlaminii* and *Fusarium solani*. The remaining isolates were represented by one species for each genus including *Paraphoma radicina*, *Coniolariella hispanica*, *Phaeoacremonium rubrigenum*, *Aspergillus* sp., *Psathyrella candolleana*, *Trichoderma asperellum*, *Alternaria chlamydosporigena*, *Thielavia microspore*, *Acremonium sclerotigenum*, *Paecilomyces lilacinus*, *Monocillium ligusticum*, and *Simplicillium cylindrosporum*.

**TABLE 1:** Overall occurrence of *Salvia abrotanoides*-colonizing fungi in plant samples collected in the Zoshk, Kalat, and Darrud areas in Iran.

	**Occurrence**		
**Taxonomic assignment of**			
**fungal isolates**	**Zoshk**	**Kalat**	**Darrud**
*Penicillium canescens*	+	+	−
*Penicillium chrysogenum*	+	−	−
*Penicillium charlesii*	+	+	−
*Penicillium murcianum*	−	+	−
*Penicillium* sp.	+	+	−
*Talaromyces verruculosus*	+	+	−
*Talaromyces* sp.	+	+	−
*Fusarium dlaminii*	−	−	+
*Fusarium solani*	−	+	−
*Paraphoma radicina*	+	−	−
*Coniolariella hispanica*	−	+	−
*Phaeoacremonium rubrigenum*	+	−	−
*Aspergillus* sp.	+	−	−
*Psathyrella candolleana*	+	−	−
*Trichoderma asperellum*	+	−	−
*Alternaria chlamydosporigena*	+	−	−
*Thielavia microspora*	+	−	−
*Acremonium sclerotigenum*	+	−	−
*Paecilomyces lilacinus*	−	−	+
*Monocillium ligusticum*	−	−	+
*Simplicillium cylindrosporum*	−	−	+

*The presence of a distinct isolate is indicated with “+,” while “−” indicates the absence of a isolate in the specified areas.*

### Secondary Metabolite Profiles

Phytochemical screening of fungus-derived ethyl acetatic extracts showed high chemical diversity of various secondary metabolites. Among the isolates, the genera *Penicillium*, *Talaromyces*, *Fusarium*, *Paraphoma* and *Coniolariella* produced the highest diversity of compounds, including terpens, fatty acid amids, vitamins, dicarboxylic acids, isoflavons, ketons, alcohols, phenols, lipids, alkaloids, catecholamins, and polyketides. The results of the qualitative assessment of phytochemical profiles of two prevalent fungal genera are shown in Table 2. They indicated that different species of *Penicillium* produce different profiles of secondary metabolites. suberic acid and pantothenic acid were the only two compounds that were produced by all *Penicillium* isolates (Table 2). Compounds that were produced by four *Penicillium* isolates included pyridoxine (*P. canescens*, *P. chrysogenum*, *P. charlesii* and *P. murcianum*), hexadecanamide (*P. canescens*, *P. chrysogenum*, *P. charlesii* and *Penicillium* sp.) and nicotinic acid (*P. canescens*, *P. charlesii*, *P. murcianum* and *Penicillium* sp.). In addition, azelaic acid and arabitol were were produced by three distinct *Penicillium* strains (*P. canescens*, *P. charlesii*, and *Penicillium* sp.). Compounds that were produced by two *Penicillium* isolates included cryptotanshinone (*P. canescens* and *P. murcianum*), manitol (*P. canescens* and *P. charlesii*), glutaric acid (*P. canescens* and *P. murcianum*), monoolein (*P. canescens* and *P. chrysogenum*) and paracetamol (*P. chrysogenum* and *Penicillium* sp.). The results revealed that indol-3-acetic acid, daidzein and nipecotic acid were produced by *P. canescens*, as well as itaconic acid and N-acetylanthranilic acid that were identified for *P. murcianum* and *Penicillium* sp., respectively. *Talaromyces* spp. produced compounds from the same chemical groups as *Penicillium* spp. (Table 2). Phenenthylamin, solanidine, and trigonelline which are all alkaloids, caffeic acid from the phenols group, grisoeofulvin (polyketides group), glycitein (isoflavones group), N-acetyldopamine (catecholamines group), acetophenone (ketones group), mevalonolactone (terpens group), and xylitol from the alcohols group were secondary metabolites produced by the *Talaromyces* isolates that differed from the profiles of *Penicillium* species. Two species within the *Fusarium* genus obtained from two different sampling sites showed substantially different profiles (Supplementary Figure S1A). Only *Fusarium dlaminii* from Darrud area produced stachydrine (alkaloid) that was specific for this species among other isolates. Except of mandelic acid, the remaining compounds produced by *Paraphoma radicina* were also common within the other isolates (Supplementary Figure S1B). Metabolic profiles of *Coniolariella hispanica* indicated that it produced secondary metabolites with less diversity; nevertheless cryptotanshinone was detected in the diterpenes group (Supplementary Figure S1C). Various isolates assigned to the genera *Penicillium* and *Talaromyces* were isolated from the sampling sites Kalat and Zoshk und thus subjected to a complementary comparison of secondary metabolite profiles. Isolates belonging to the same species shared 25–57% of the identified secondary metabolites in case of *Penicillium* and 23 – 40% in case of *Talaromyces* (Supplementary Table S1).

**TABLE 2:** Secondary metabolite profiles of endophytic fungal isolates assigned to *Penicillium* spp. and *Talaromyces* spp. that were identified in the cultivation medium.

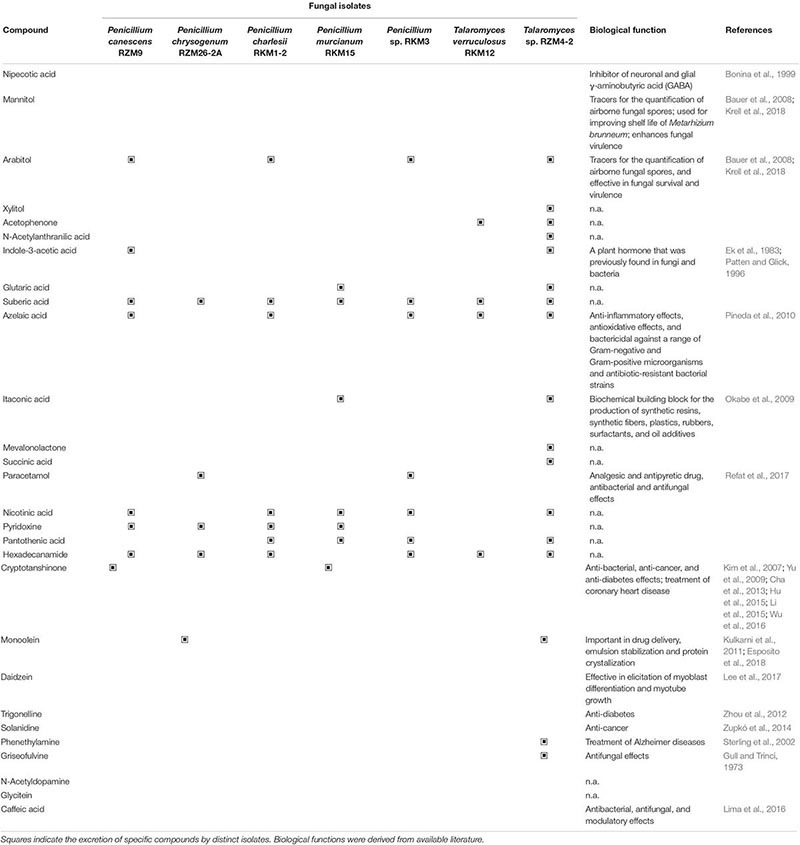

*Squares indicate the excretion of specific compounds by distinct isolates. Biological functions were derived from available literature.*

**FIGURE 1:**
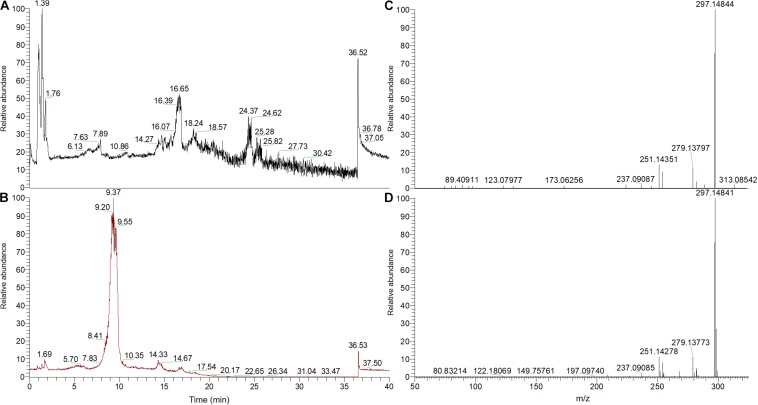
Cryptotanshinone detection with high-resolution mass spectrometry. *Penicillium murcianum* extracts were compared to a 50 ppm cryptotanshinone standard. **(A,B)** Total ion chromatograms of the *Penicillium murcianum* extract and the cryptotanshinone standard, respectively. **(C,D)** High-resolution MS2 product ions of the *Penicillium murcianum* extract and the cryptotanshinone standard, respectively. The chromatograms and high-resolution MS2 product ion spectra of the other isolates are included as Supplementary Figures S2, S3).

Moreover, LC-MS analyses showed that *Penicillium canescens*, *P. murcianum*, *Paraphoma radicina*, and *Coniolariella hispanica* can produce cryptotanshinone independent of the host plant (Figure 1 and Supplementary Figures S2, S3). Analysis of the secondary metabolite profile of the host plant showed that azelaic acid and suberic acid (data not shown), which are dicarboxylic acids as well as cryptotanshinone were present in host plant extracts. The overall results of the phytochemical analysis indicated that endophytic fungi from *S. abrotanoides* can produce several phytochemicals that are either identical or structurally similar to those of the host plant, as well as new bioactive compounds that are not present in the host plant.

### Effects of Exogenous GA_3_ on Cryptotanshinone Production in *S. abrotanoides* and the Isolated Fungi

**FIGURE 2:**
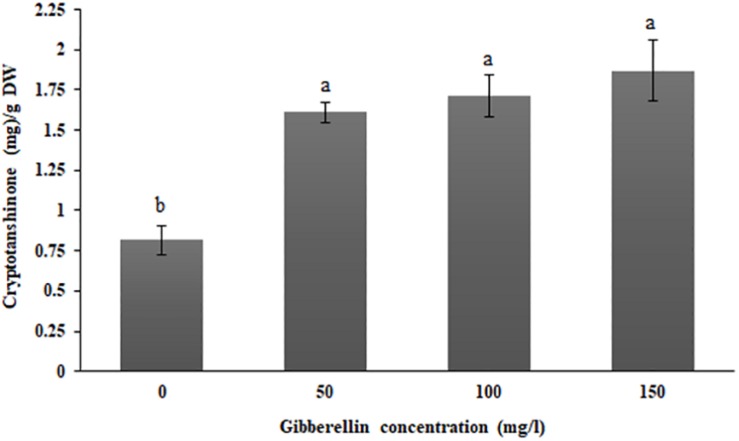
GA_3_ treatment-induced increase the cryptotanshinone biosynthesis in *S. abrotanoides* plants. Treatments with sterile water without GA_3_ supplementation (0 mg l^–1^) were considered as controls. Each value represents the mean ± standard error of three replicates; letters (a and b) indicate significant differences at *P* < 0.05. The experiments were conducted with three biological replicates for each tested concentration.

In order to explore the effect of GA_3_ supplementation on cryptotanshinone biosynthesis in *S. abrotanoides*, exogenous GA_3_ was supplemented in three different concentrations. Spray application of sterile water without GA_3_ was implemented as a control for comparative assessments. Analytical quantifications showed that the GA_3_-treated plants responded with a significant increase in cryptotanshinone biosynthesis in comparison to the control (Figure 2). When 50 mg l^–1^ GA_3_ were sprayed on *S. abrotanoides* plants, the cryptotanshinone concentration in the plant increased to 1.60 ± 0.06 mg g^–1^. In the 100 mg l^–1^ GA_3_ treatment, the cryptotanshinone concentration increased to 1.71 ± 0.13 mg g^–1^, while at the highest applied concentration of 150 mg l^–1^, the concentration increased to 1.87 ± 0.19 mg g^–1^.

**FIGURE 3:**
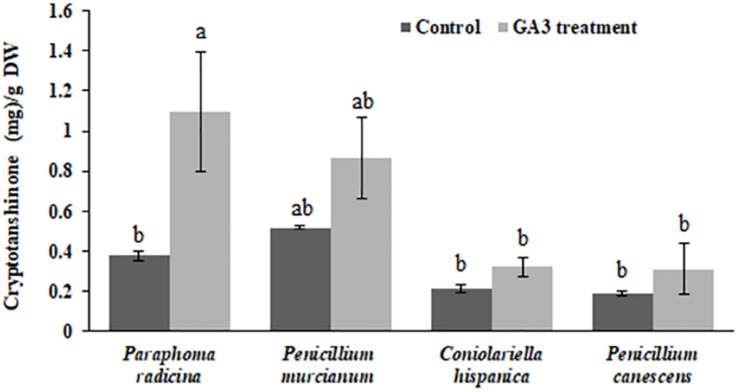
Increase of fungal cryptotanshinone production after GA_3_ treatments. Each isolate without GA_3_ treatment was considered as a control. Each value represents the mean ± standard error of three replicates; small letters (a and b) indicate significant differences at *P* < 0.05. The experiments were conducted with three biological replicates for each isolate.

In addition to the exogenous hormonal applications to *S. abrotanoides* plants, the effect of 50 mg l^–1^ GA_3_ on each fungus producing cryptotanshinone was investigated. Analytical quantifications showed an increase in the amount of cryptotanshinone production by the fungi after treatment with GA_3_ (Figure 3). When GA_3_ at a concentration of 50 mg l^–1^ was supplemented to *Paraphoma radicina* cultures, the cryptotanshinone concentration showed a significant increase from 0.37 ± 0.02 mg g^–1^ in the control to 1.09 ± 0.29 mg g^–1^ for the GA_3_ treatment. Moreover, when GA_3_ was supplemented at a concentration of 50 mg l^–1^ to different fungal isolates, again an increase in cryptotanshinone production was observed, however, the significance was not confirmed with statistical analyses. For *Penicillium murcianum*, *Coniolariella hispanica* and *Penicillium canescens*, the cryptotanshinone concentration increased to 0.86 ± 0.2 mg g^–1^ (0.51 ± 0.008 mg g^–1^ in the control), 0.23 ± 0.04 mg g^–1^ (0.21 ± 0.01 mg g^–1^ in the control), and 0.31 ± 0.12 mg g^–1^ (0.19 ± 0.01 mg g^–1^ in the control), respectively.

## Discussion

In the present study, different arid areas in Northern Iran were selected to investigate secondary metabolite profiles of endophytic fungi isolated from indigenous *S. abrotanoides* plants. All *Salvia* plants harbored taxonomically distinct fungal endophytes, especially in their roots, that produced an unexpectedly broad spectrum of secondary metabolites. The results revealed isolate-specific secondary metabolite profiles, e.g., illustrated by distinct spectra obtained with *Penicillium* isolates. We identified a broad range of well-studied fungal secondary metabolites as well as such that were not yet described in fungi, e.g., cryptotanshinone a major bioactive diterpenoid previously isolated from *Salvia* species.

The untargeted profiling approach of secondary metabolites in the isolated plant endophytes resulted in the detection of a broad spectrum of compounds from various chemical groups. Compounds including itaconic acid and azelaic acid that were detected in *Penicillium* and *Talaromyces* isolates, were previously also detected in isolates assigned to the genus *Aspergillus* and connected to plant-microbe interactions (Okabe et al., 2009; Pineda et al., 2010). In the present study, only a low diversity of secondary metabolites were detected in the *Aspergillus* isolate (data not shown) and none of the two organic acids was present. Itaconic acid was shown to have a high potential as a biochemical building block for the production of synthetic resins, synthetic fibers, plastics, rubbers, surfactants, and oil additives (Okabe et al., 2009). Azelaic acid has profound anti-inflammatory, antioxidative effects, and is bactericidal against a range of Gram-negative and Gram-positive microorganisms as well, including antibiotic-resistant bacterial strains (Pineda et al., 2010). Most of the isolates produced a variety of sugar alcohols. Sugar alcohols including arabitol and mannitol that are considered as tracers for the quantification of airborne fungal spores (Bauer et al., 2008), improve survival of fungi under drought conditions, and shelf life of encapsuled *Metarhizium brunneum* coupled with enhanced fungal virulence (Krell et al., 2018). *Penicillium canescens* was shown to produce nipecotic acid, a piperidinemonocarboxylic acid that belongs to beta-amino acids. Nipecotic acid is one of the most potent inhibitors of neuronal and glial γ-aminobutyric acid (GABA) and thus relevant for medicinal applications (Bonina et al., 1999). *Penicillium canescens*, two species of *Fusarium*, and *Paraphoma radicina* produced daidzein, a natural isoflavone known from the *Leguminosae* plant family. This metabolite has been shown to elicit myoblast differentiation and myotube growth (Lee et al., 2017). Daidzein was previously found in *Trichoderma* sp., an endophytic fungus of *Azadirachta indica* (Xuan et al., 2014). Although *Trichoderma* spp. are common endophytes in plants, only one isolate was recovered from *Salvia abrotanoides* in the present study and diadezein was not found in its secondary metabolite profile (data not shown). Furthermore, *Penicillium canescens, Penicillium chrysogenum*, *Talaromyces* sp. and *Paraphoma radicina* were shown to produce monoolein which is considered one of the most important lipids in the fields of drug delivery, emulsion stabilization and protein crystallization (Kulkarni et al., 2011; Esposito et al., 2018). In addition, a broad range of vitamins was identified in the metabolite profiles of the endophytic fungi. Vitamins including nicotinic acid, pyridoxine and pantothenic acid were previously reported in *Fusarium proliferatum* and *Cercospora nicotianae* (Harvais and Pekkala, 1975; Wetzel et al., 2004; Jin et al., 2013). This is in accordance with the findings in the present study, where *Fusarium* isolates were found to produce three different vitamins or precursors thereof. Paracetamol (acetaminophen) was only detected in *Penicillium chrysogenum* and *Penicillium* sp. metabolite profiles. This compound is one of the most popular and most commonly used analgesic and antipyretic drugs around the world, which also had antibacterial and antifungal properties (Refat et al., 2017). Different reports show that distinct microorganisms can utilize paracetamol as a carbon and energy source (Wu et al., 2012), however, fungal biosynthesis of this compound was not described so far. The degradation capacity indicates that it might be commonly found in natural environments, where fungi could be involved in its biosynthesis. Deepening analyses are required to clarify if distinct members of the genus *Penicillium* genus produce the bioactive molecule or a structural analog thereof. Indole-3-acetic acid as a plant hormone was identified in metabolite profiles of a broad range of fungal genera that were subjected to metabolic profiling in the present study. This plant hormone was previously found in a broad range of fungi and bacteria (Ek et al., 1983; Patten and Glick, 1996). *Talaromyces* spp. profiles also showed other chemically diverse compounds including trigonelline, solanidine and phenethylamine, they are used in treatments of diabetes, cancer and Alzheimer diseases, respectively (Sterling et al., 2002; Zhou et al., 2012; Zupkó et al., 2014). *Talaromyces* sp. and *Paraphoma radicina* produced mandelic acid, which is a monocarboxylic acid. Mandelic acid has been used as an antibacterial agent, particularly in the treatment of urinary tract infections (Putten, 1979). Recently, mandelic acid production was achieved by microbial fermentations using engineered *Escherichia coli* and *Saccharomyces cerevisiae* expressing heterologous hydroxymandelate synthases (Reifenrath et al., 2018). Moreover, caffeic acid was found in metabolite profiles of isolates assigned to the genera *Talaromyces* and *Paraphoma*. This well-known phenol has been previously found in endophytic fungi *Cladosporium velox* (Singh et al., 2016), *Penicillium canescens* and *Fusarium chlamydosporum* (Das et al., 2018). Antibacterial, antifungal and modulatory effects of caffeic acid have been shown in previous studies (Lima et al., 2016). *Talaromyces sp*. was the only isolate that produced griseofulvin that is a polyketide. Griseofulvin is an antifungal antibiotic widely used for the treatment of human and animal dermatophytic infections (Gull and Trinci, 1973). This compound has been previously detected in endophytic fungi including *Penicillium griseofulvum* (Zhang et al., 2017), *Nigrospora* sp. (Zhao et al., 2012), and *Xylaria* sp. (Park et al., 2005). Our results indicate that fungal isolates from *S. abrotanoides*, but different geographic isolation sources can have differing secondary metabolite profiles even if they belong to the same species. In this context, it must be taken into account that these isolates would likely show genetic differences when assessed at the strain level. Previous studies that addressed other plant-endophyte systems have shown that geographic as well as seasonal differences influence endophytic communities (Collado et al., 1999; Christian et al., 2016).

In general, the plant-endophytic isolates were shown to produce a broad variety of exploitable metabolites, however, one particularly important compound, that was found to be also synthetized during axenic fermentation approaches in the present study, was cryptotanshinone. Previous studies showed that cryptotanshinone has antibacterial activity (Lee et al., 1999), inhibits angiogenesis (Hur et al., 2005) and inhibits STAT3 in prostate cancer (Shin et al., 2009). Moreover, it was previously reported that cryptotanshinone can be used for the treatment of coronary heart disease (Yu et al., 2009) and diabetes (Kim et al., 2007). These promising medicinal properties, as well as the general demand for bioactive compounds that are produced under controlled settings, led us to explore possibilities to increase cryptotanshinone production in *S. abrotanoides* and its endophytic fungi. Previous studies have shown that there is an overlap between the tanshinone and the gibberellin biosynthesis pathways (Su et al., 2016). Detailed assessments showed two distinctive GA biosynthesis pathways are present in plants and fungi that have the copalyl diphosphate synthase and kaurene synthase enzymes in common (Hedden et al., 2001). Copalyl diphosphate synthase and kaurene synthase are also the main enzymes in the tanshinone biosynthetic pathway (Gao et al., 2014). We expected that supplementation of one end product might regulate conversion pathways in favor of the other compound. Therefore, we explored the potential of exogenous gibberellin supplementation to increase cryptotanshinone production by the plant and isolated endophytic fungi. The present study demonstrated that GA_3_ significantly increases the amount of cryptotanshinone in *S. abrotanoides*. This finding is in consensus with reports that have shown gibberellins have been effective in increasing the levels of tanshinones in *Salvia miltiorrhiza* (Yuan et al., 2008). Moreover, our finding showed an increase in cryptotanshinone production in different endophytic fungi when compared when they were cultivated under laboratory conditions. The overall findings provide an exploitable basis for the cultivation of *S. abrotanoides* plants with higher concentrations of its bioactive compounds and for biotechnological applications based on its endophytes.

## Data Availability Statement

The datasets generated for this study can be found in the GenBank under accession numbers MK367721–MK367776.

## Author Contributions

JA, AG, and YT-B designed the study. YT-B carried out the plant collection and fungi isolation under the supervision of AG. YT-B carried out the molecular identification of fungi, LC-MS, secondary metabolite profiles, GA_3_ experiments, and analyzed the LC-MS data. HM and TC supervised the molecular identification and GA_3_ experiments. AG subjected the GA_3_ experiments data to statistical analyses. YT-B and TC wrote the final version of the manuscript. GB reviewed the final version of the manuscript. All authors read and approved the final version of the manuscript.

## Conflict of Interest

The authors declare that the research was conducted in the absence of any commercial or financial relationships that could be construed as a potential conflict of interest.

## Footnotes

1 https://www.mzcloud.org

## Abbreviations

GA_3_: gibberellin 3
HPLC-MS: high-performance liquid chromatography-mass spectrometry
ITS: internal transcribed spacer region
LC-MS: liquid chromatography-mass spectrometry
LSD: Least Significant Difference
MA: malt extract agar
PGB: potato glucose broth.
