# Supplementary material for: Endophytic Fungi of Native Salvia abrotanoides Plants Reveal High Taxonomic Diversity and Unique Profiles of Secondary Metabolites
Source: Front Microbiol. 2020 Jan 17;10:3013. doi: 10.3389/fmicb.2019.03013 (PMC6978743; doi:10.3389/fmicb.2019.03013)
Supplement: Supplementary file 1 [file Data_Sheet_1.docx]

**Supplementary material**

**Endophytic fungi of native *Salvia abrotanoides* plants reveal high taxonomic diversity and unique profiles of secondary metabolites**

Running title: Secondary metabolites of *S*. *abrotanoides* endophytes

**Yeganeh Teimoori-Boghsani^1^, Ali Ganjeali^1^, Tomislav Cernava^3*^, Henry Müller^3^, Javad Asili^2^, and Gabriele Berg^3^**

^1^Department of Biology, Faculty of Science, Ferdowsi University of Mashhad, Mashhad, Iran

^2^Department of Pharmacognosy, School of Pharmacy, Mashhad University of Medical Sciences, Mashhad, Iran

^3^Institute of Environmental Biotechnology, Graz University of Technology, Petersgasse 12, 8010 Graz, Austria

***Correspondence:**

Tomislav Cernava,

Institute of Environmental Biotechnology,

Graz University of Technology, Petersgasse 12,

8010 Graz, Austria.

e-mail: tomislav.cernava@tugraz.at

**Keywords:** *Salvia abrotanoides*, Endophytic fungi, Secondary metabolites, Cryptotanshinone, Gibberellin

**Table S1**. Secondary metabolite profiles of endophytic fungal isolates assigned to *Penicillium spp*. and *Talaromyces spp*. from different sampling sites.

|  | *Penicillium canescens* | | *Penicillium charlesii* | | *Penicillium sp.* | | *Talaromyces verruculosus* | | *Talaromyces sp.* | |
| --- | --- | --- | --- | --- | --- | --- | --- | --- | --- | --- |
| Compound | Kalat | Zoshk | Kalat | Zoshk | Kalat | Zoshk | Kalat | Zoshk | Kalat | Zoshk |
| **Nipecotic acid** |  | 🞕 |  |  |  |  |  |  |  |  |
| **Mannitol** | 🞕 | 🞕 | 🞕 |  |  |  |  |  |  |  |
| **Arabitol** |  | 🞕 | 🞕 |  | 🞕 |  |  |  |  | 🞕 |
| **Xylitol** |  |  |  |  |  |  |  |  |  | 🞕 |
| **Acetophenone** |  |  |  |  |  |  | 🞕 |  |  | 🞕 |
| **N-Acetylanthranilic acid** |  |  |  |  | 🞕 |  |  |  |  | 🞕 |
| **Indole-3-acetic acid** |  | 🞕 |  |  |  |  |  |  |  | 🞕 |
| **Glutaric acid** | 🞕 | 🞕 |  |  |  |  |  |  | 🞕 | 🞕 |
| **Suberic acid** | 🞕 | 🞕 | 🞕 | 🞕 |  | 🞕 | 🞕 | 🞕 |  | 🞕 |
| **Azelaic acid** |  | 🞕 | 🞕 |  | 🞕 |  | 🞕 |  | 🞕 | 🞕 |
| **Itaconic acid** |  |  |  |  |  |  |  |  |  | 🞕 |
| **Mevalonolactone** |  |  |  |  |  |  |  |  |  | 🞕 |
| **Succinic acid** |  |  |  |  |  |  |  |  | 🞕 | 🞕 |
| **Paracetamol** |  |  |  |  | 🞕 |  |  |  |  |  |
| **Nicotinic acid** | 🞕 | 🞕 | 🞕 | 🞕 | 🞕 | 🞕 |  |  |  | 🞕 |
| **Pyridoxine** | 🞕 | 🞕 | 🞕 |  |  |  |  |  |  |  |
| **Pantothenic acid** | 🞕 | 🞕 | 🞕 |  | 🞕 | 🞕 |  |  | 🞕 | 🞕 |
| **Hexadecanamide** |  | 🞕 | 🞕 |  | 🞕 | 🞕 | 🞕 |  |  | 🞕 |
| **Cryptotanshinone** | 🞕 | 🞕 |  |  |  |  |  |  |  |  |
| **Monoolein** |  | 🞕 |  |  |  |  |  |  |  | 🞕 |
| **Daidzein** |  | 🞕 |  |  |  |  |  |  |  |  |
| **Trigonelline** |  |  |  |  |  |  |  |  |  | 🞕 |
| **Solanidine** |  |  |  |  |  |  |  |  |  | 🞕 |
| **Phenethylamine** |  |  |  |  |  |  |  |  |  | 🞕 |
| **Griseofulvine** |  |  |  |  |  |  |  |  |  | 🞕 |
| **N-Acetyldopamine** |  |  |  |  |  |  |  |  |  | 🞕 |
| **Glycitein** |  |  |  |  |  |  |  |  |  | 🞕 |
| **Caffeic acid** |  |  |  |  |  |  | 🞕 | 🞕 | 🞕 | 🞕 |


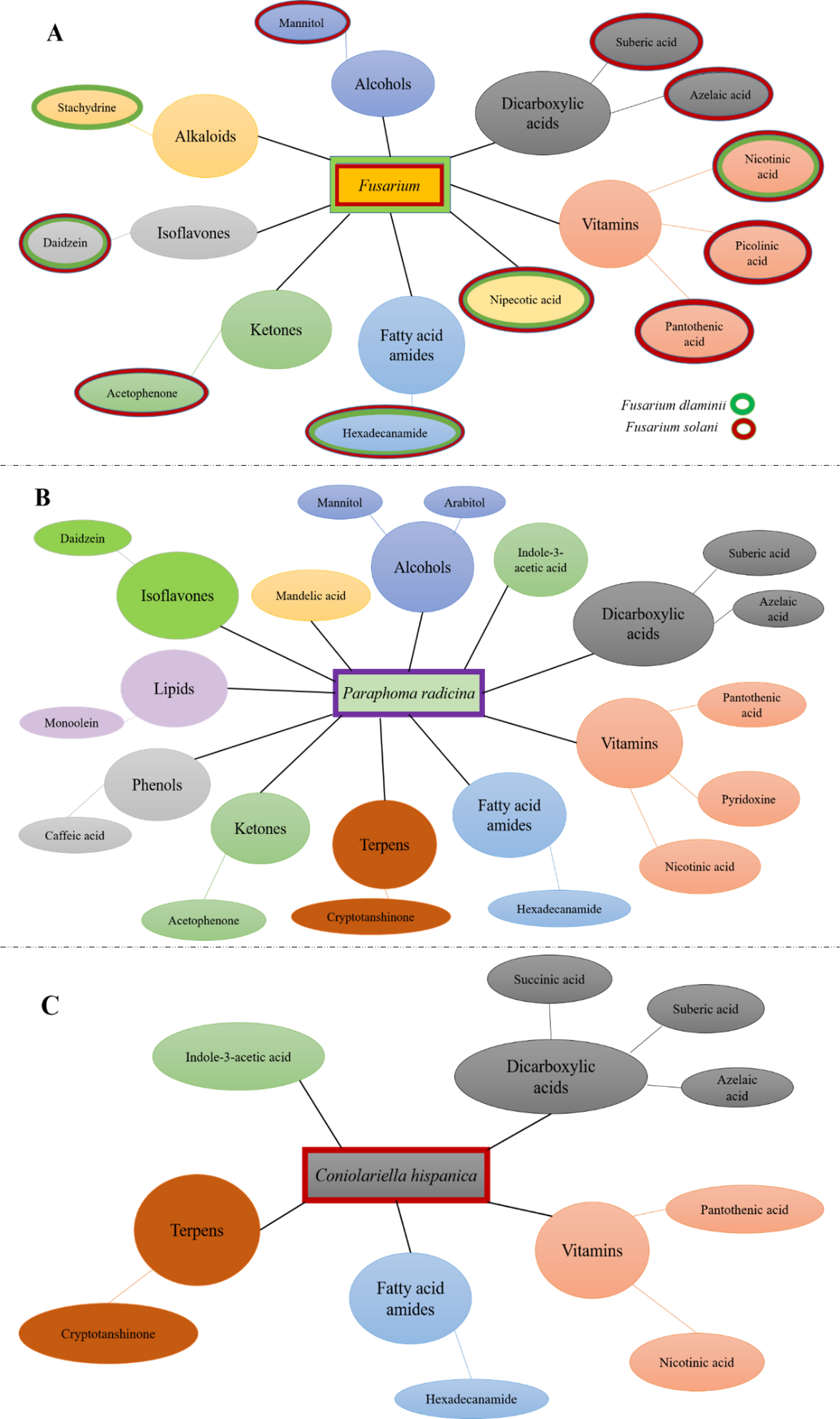


**Figure S1.** Schematic profiles of secondary metabolites produced by fungal endophytes. For *Fusarium* species (A), the color rings indicate the occurrence of each compound in either *F. dlaminii* or *F. solani*. The genera *Paraphoma* (B) and *Coniolariella* (C) were represented by one species respectively.


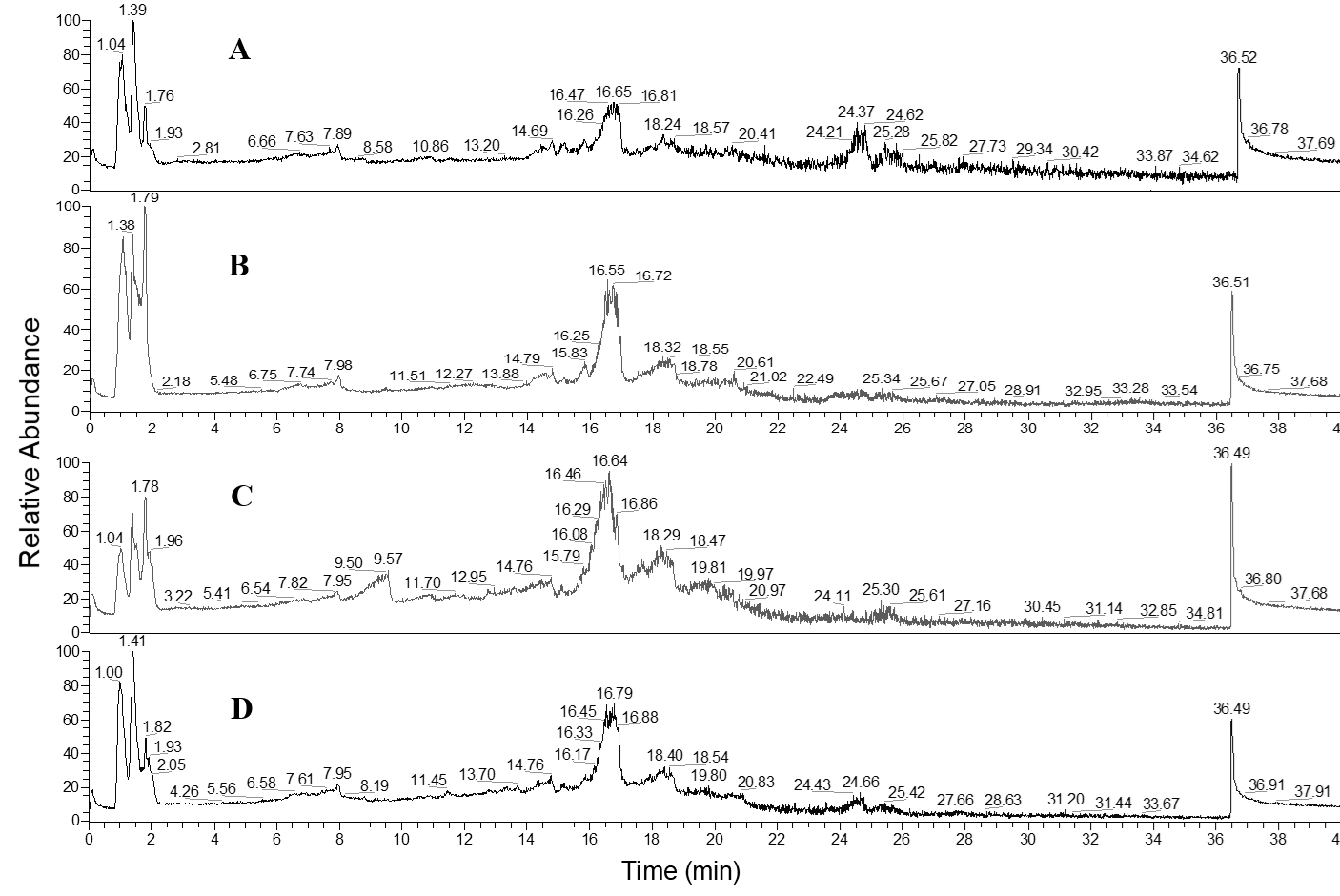


**Figure S2.** Total ion chromatograms of *Penicillium murcianum* (A), *Coniolariella hispanica* (B), *Paraphoma radicina* (C), and *Penicillium canescens* (D).


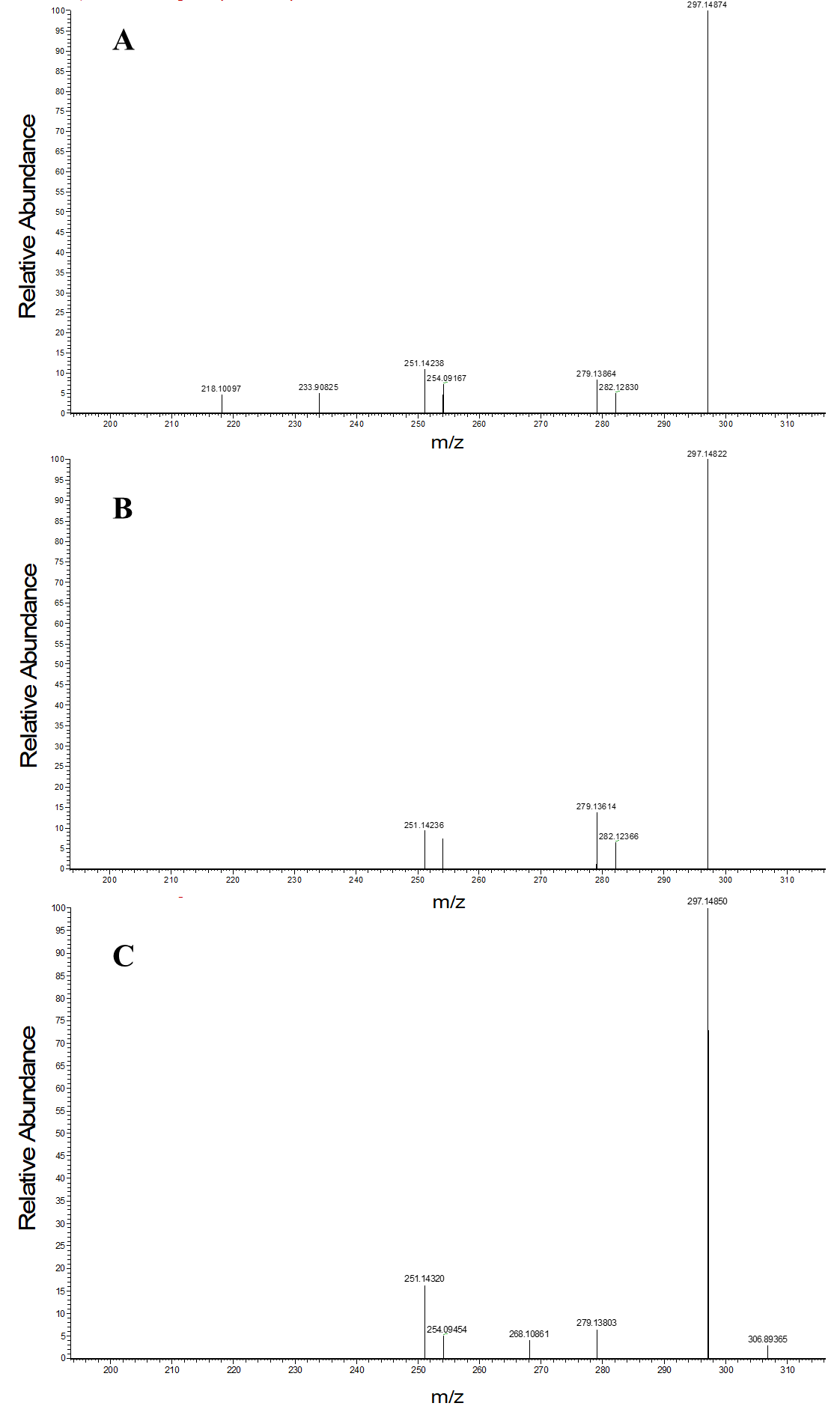


**Figure S3.** High-resolution MS2 product ions of *Coniolariella hispanica* (A), *Paraphoma radicina* (B), and *Penicillium canescens* (C).
